# Demographic analysis of a low resource, socioculturally diverse urban community presenting for infertility care in a United States public hospital

**DOI:** 10.1186/s40834-017-0044-7

**Published:** 2017-05-03

**Authors:** Jacqueline R. Ho, Jacquelyn R. Hoffman, Lusine Aghajanova, James F. Smith, Marisela Cardenas, Christopher N. Herndon

**Affiliations:** 1 Obstetrics & Gynecology, University of Southern California, 2020 Zonal Ave, Los Angeles, CA 90033 USA; 2 Obstetrics, Gynecology, & Reproductive Sciences, University of California, 550 16th Street, San Francisco, CA 94158 USA; 3 Department of Urology, University of California, 400 Parnassus, Box 0738, San Francisco, CA 94143 USA; 4 UCSF Philip R. Lee Institute for Health Policy Studies, 3333 California St, San Francisco, CA 94118 USA; 5 University of California, 101 Sproul Hall, Berkeley, CA 94704 USA; 6 Alta Bates IVF Program, 2999 Regent St Suite 101A, Berkeley, CA 94705 USA

**Keywords:** Infertility, Access to care, Underserved, Immigrant

## Abstract

**Background:**

Infertility is a prevalent disease of reproductive health that exerts an impact on an estimated 80 million people worldwide. For many, involuntary childlessness becomes a central and preoccupying issue in their lives, the impact of which is exacerbated by lack of access to basic care and treatment. These effects maybe further magnified among immigrant communities, a growing but highly marginalized population that has been shown in other areas of reproductive health to experience worse health outcomes and delays in access to care. To date, few studies have examined the unique medical and sociocultural considerations of infertility among immigrant populations in the United States.

**Methods:**

Our study is a cross-sectional analysis of women presenting for infertility evaluation at a county hospital serving a low resource, socioculturally diverse largely immigrant communities in comparison to infertile women from a largely affluent population presenting to a high resource, comprehensive fertility center. We employed surveys to evaluate demographics and socioeconomic parameters as well as abstracted data from medical records to obtain infertility diagnoses. Multivariate regression analysis was applied to examine impact of sociocultural factors as predictors of duration of untreated infertility disease burden experienced by patients.

**Results:**

Eighty-seven women were included in our analysis. In the county hospital/low resource clinic (LR), the mean age was 32.9 ± 4.9 vs 36.4 ± 6.3 years in the fee-for-service/high resource clinic (HR). The mean reported duration of infertility in LR and HR patients was 3.4 ± 3.0 vs 2.3 ± 1.5 years. 70% of LR patients were monolingual non-English speakers vs 5.4% of HR patients. 59% of LR patients reported an annual household income of less than $25,000 and 70% did not have a college degree. 81.1% of HR patients reported an income of higher than $100,000, and 81.1% had completed college or graduate school. The most common infertility diagnosis in the LR was anovulation (38%) and tubal factor (28%) compared to diminished ovarian reserve (37.8%) and male factor (51.4%) in the HR. After controlling for age at the initiation of pregnancy attempt, lower education level, lower income, and immigrant status were significantly correlated with a longer duration of infertility.

**Conclusions:**

Women presenting for infertility care to a low resource county medical center represent immigrant communities and are generally of younger age, but with a longer duration of infertility. This study identifies lower educational level, income, and immigrant status as barriers in access to care.

**Electronic supplementary material:**

The online version of this article (doi:10.1186/s40834-017-0044-7) contains supplementary material, which is available to authorized users.

## Background

An estimated 70–80 million couples in the world suffer from involuntary childlessness [1]. Psychosocial consequences of infertility can be severe, including social isolation and domestic violence [2, 3]. Affected women often have higher rates of depression and reduced sexual satisfaction [4]. In developing nations, infertility has significant implications on quality of life, but the resources for the workup and treatment are globally limited [5, 6]. In countries with the largest populations, in vitro fertilization (IVF) is offered to <1% of those in need, and in developing nations is virtually nonexistent [7]. In developed nations, assisted reproductive technologies (ART) are widely available but are predominately concentrated around urban centers [8]. Nonetheless, disparities in access to infertility care remain staggering, particularly in the United States (US). In an economic analysis published in 2009, only 24% of the demands for ART in the US were met. The average cost of an IVF cycle was highest in the US and comprised the highest percentage of gross national income per capita [9].

Numerous studies have established biologic disparities in outcome among ethnic minorities, particularly in regards to ART outcomes [10–13]. Less is known about the disparities and socioeconomic factors impacting infertility in these groups. This population is relevant given the trend in rising immigrant population, which was estimated to comprise 40 million individuals (13% of the total population) in the US in 2010 [14]. Pioneering studies by Becker and Nachtigall qualitatively examined the infertility experience of a largely immigrant Latino population presenting for infertility care at an urban county general hospital. They found that income, insurance status, language and cultural barriers, and bureaucracy within the public health system created challenges in accessing appropriate infertility resources [15, 16].

While there is some knowledge about social factors affecting access to care, no study to date has examined the infertility demographic of patients from low resource, socioculturally diverse, largely immigrant communities presenting for infertility care in the US. Understanding the causes of infertility and sociocultural factors of this population is essential for developing treatment strategies to address the untreated disease burden. In our study, we sought to compare the demographics, socioeconomic characteristics, and causes of infertility in patients seeking infertility treatment in a low resource clinic with patients presenting a fee-for-service comprehensive fertility clinic in the same urban setting. We examined impact of sociocultural factors as predictors of duration of untreated infertility disease burden experienced by patients.

We hypothesized that a lack of English proficiency, immigrant status, and a lower income and education would be associated with increased duration of infertility.

## Methods

### Recruitment and study population

We recruited women presenting for initial infertility treatment in two university-affiliated Reproductive Endocrinology and Infertility (REI) clinics from 2012 to 2014 in San Francisco, California. The public county-based low resource clinic (LR) for uninsured patients, San Francisco General Hospital (SFGH), is publicly funded and provides free and low cost medical care to a largely immigrant, culturally diverse, and indigent population. The REI clinic operates once weekly, offering basic diagnostic workup and treatment for patients. Clomiphene citrate is utilized for ovulation induction in anovulatory patients and for superovulation in patients with unexplained infertility. Intrauterine insemination, superovulation with exogenous gonadotropins, and ART services are not available. Medical students and residents training in Obstetrics and Gynecology staff the clinic and are supervised by a board certified Reproductive Endocrinologist. The fee-for-service, high resource infertility clinic (HR) was at the University of California San Francisco Center for Reproductive Health (UCSF), a large comprehensive tertiary fertility care center. We included all women ≥18 years old seeking initial evaluation for infertility. There were no exclusion criteria. Approval for this research project was obtained from the committee on human research at SFGH and UCSF; written informed consent was obtained from all participants.

### Infertility evaluation

Infertility was defined as the inability to conceive after twelve months of regular intercourse for women < 35 years old or six months for women ≥ 35 years old [17]. Duration of infertility was defined as the amount of time passed between the age at attempting pregnancy and the initial evaluation for infertility. At each clinical site, evaluation included a focused history and physical exam. The minimum laboratory tests ordered for workup included thyroid stimulating hormone, prolactin, cycle day 2 or 3 follicular stimulating hormone (FSH) and estradiol, antimüllerian hormone (AMH), luteal progesterone, semen analysis, and hysterosalpingogram. Random FSH, luteinizing hormone, and estradiol were drawn for patients with a history of oligomenorrhea. Pelvic ultrasound and saline infusion sonogram was also performed as clinically indicated; due to limited resources and time constraints, pelvic ultrasound could not be routinely performed on LR patients. Pelvic ultrasound with assessment of antral follicle count (AFC) was routinely performed on each patient at the HR clinic. Laboratory and radiographic data were interpreted as appropriate by a reproductive endocrinologist to determine the etiology of infertility. Diagnoses were broken down into the following categories: diminished ovarian reserve (DOR), anovulation, tubal factor, uterine factor, male factor, recurrent pregnancy loss, and unexplained infertility, defined by America Society of Reproductive Medicine (ASRM) diagnostic criteria [18]. DOR was defined as having at least one of the following: day 3 FSH >10 IU/L, AMH <1 ng/ml, or lower than expected number of antral follicles for age. At the LR clinic, AFC was not routinely performed, so either basal FSH or AMH were used to establish the diagnosis of diminished ovarian reserve. Anovulation was defined by report of irregular menses and/or properly timed midluteal progesterone < 3 ng/ml. Tubal factor was defined as the presence of hydrosalpinges, salpingitis isthmica nodosa, history of salpingectomy, or bilateral tubal occlusion due to either infection or previous bilateral tubal ligation as documented on hysterosalpingogram or surgical evaluation. Uterine factor was defined as any intrauterine pathology requiring surgical correction such as submucosal fibroids, polyps, and synechiae. Male factor was determined by an abnormal semen analysis as defined by World Health Organization (WHO) 2010 criteria [19]. Patients were given a diagnosis of unexplained infertility if previously described workup was within normal limits. Although not an etiology of infertility, recurrent pregnancy loss (RPL) was included in our descriptive analysis. RPL was defined as having three or more, or two consecutive, pregnancy losses in the past [20].

Diagnosis categories were not mutually exclusive. In patients with multifactorial infertility, each incident of cause counted towards the total number of diagnoses, for this reason the number of infertility diagnoses exceeded the number of patients.

### Demographic and socioeconomic data

Surveys were administered (Additional file 1) to obtain sociocultural data, including age, parity, duration of infertility, marital status, English proficiency, country of origin, number of years living in the US, ethnicity, annual household income, and educational level. Annual household income was broken down into seven groups. For our study we reclassified groups into: <$25,000, $25,000–$49,999, $50,000–$99,000, $100,000–$199,000, and ≥ $200,000 per year. Educational level was defined as highest level attained, including elementary school, high school/GED, some college, college, and graduate school. Ethnicity was self-reported by patients, including White, Black, Latino, Asian, mixed, and other. Non-English speaking women were surveyed in their native language with certified interpreters in person or via telephone to ascertain basic demographic, clinical, and socioeconomic information. English-speaking women were given the same survey in paper or electronic format to fill out.

Data were abstracted from medical records and the participants’ corresponding survey. Information was entered into a secure computer file with unique, anonymous identifier codes. We analyzed the database for outlying data points and used statistical software to assess the ranges and distribution of the inputs in order to verify the accuracy of data.

### Statistical analysis

Numerical data were assessed for normality. For descriptive statistics, all patients were included, and student’s *t*-test was used to evaluate differences in means. Fisher’s exact or *χ* ^2^ were used to evaluate categorical variables. We performed a bivariate analysis of sociodemographic factors on the duration of infertility using linear regression. For this analysis, women with previous infertility treatments were excluded (*n* = 2). The outcome was the reported duration of infertility modeled as a continuous variable. Predictor variables were chosen a priori based on clinical relevance and included English proficiency, nulliparity, ethnicity, immigrant status, income level and education level. Due to low numbers, we repeated the analysis dichotomizing income into less than $100,000 and greater than or equal to $100,000. For the same reason, education level was dichotomized to those that never attended college and those that did attend college. We counted immigrants as those arriving to the US after age 18, as we assumed that the duration of time spent in the US in childhood or adolescence was not related to infertility status and outcomes. For immigrants, linear regression was used to examine the relationship between number of years lived in the US (after age 18) with duration of infertility. For all analyses, we adjusted for women’s age at the start of pregnancy attempt, as women ≥35 are advised to seek infertility workup and treatment after 6 months rather than 1 year.

A multivariable linear regression model was utilized to analyze the relationship between sociodemographic factors and duration of infertility. We used predictor variables with *p* ≤ 0.25 at bivariate analysis to construct the model, which included English proficiency, income, education level, immigrant status, and age at the initiation of pregnancy attempt.

All *p* values were based on two-tailed tests, with statistical significance indicated by *p* < 0.05 (95% confidence interval excluding zero for regression models). STATA 13 (Statacorp, College Station, TX, USA) was used for all analyses.

## Results

Eighty-seven patients agreed to participate and were consented for the study, with 50 women from the LR clinic and 37 women from the HR clinic. LR patients had a mean age of 32.9 ± 4.1 years (range 20–44), while HR patients had a mean age of 36.4 ± 6.3 years (range 28–44) (*p* = 0.005). LR patients’ mean reported duration of infertility was 3.4 ± 2.8 years compared to 2.3 ± 1.5 years for HR patients (*p* = 0.045).

At the LR clinic, 35/50 (70%) patients were primarily non-English speakers, compared to the HR clinic, where 35/37 (94.6%) of patients were proficient in English (*p <* 0.001)*.* In the LR clinic, 50/50 (100%) patients vs 4/37 (10.8%) in the HR clinic immigrated to the US after 18-years old. There were significant differences in income and educational levels between groups. In the LR population, 29/50 (58%) reported an annual combined household income of less than $25,000 per year vs 1/37 (2.7%) in the HR group (*p <* 0.001). This is close to the federal poverty line, which is defined as $15,730 for 2 people, $19,790 for 3 people, and $23,850 for 4 people [21]. Meanwhile, HR patients 29/37 (78.4%) reported having an annual household income of higher than $100,000 vs 0/50 (0%) of the LR group (*p <* 0.001). Additionally, 35/50 (70%) of LR patients did not have a college degree compared to 7/37 (18.9%) of the HR group (*p* = 0.03, *p* < 0.001) (Table 1).

**Table 1:** Patient demographics and socioeconomic parameters

		Low resource clinic (*n* = 50)	High resource clinic (*n* = 37)	*P*-value
Characteristics of Participants				
Age (years)^a^		32.9 ± 4.1	36.4 ± 6.3	0.005^c^
Parity^b^	Nulliparity	18 (36)	31 (83.7)	<0.001^d^
	Multiparity	32 (64)	6 (16.2)	<0.001^d^
Duration of infertility (years)^a^		3.4 ± 2.8	2.3 ± 1.5	0.045^c^
Previous infertility treatments^b^		0 (0%)	2 (5.4%)	0.1
Duration of time lived in US (years)		15 ± 1.89	29.4 ± 1.7	<0.001^c^
Socioeconomic Data				
Household income^b^				
<$25,000/year		29 (58%)	1 (2.7%)	<0.001^b^
$25,000–$49,999/year		14 (28%)	0 (0%)	<0.001^b^
$50,000–99,000/year		1 (2%)	5 (13.5%)	0.44
$100,000–$199,999/year		0 (0%)	17 (45.9%)	<0.001^b^
≥$200,000/year		0 (0%)	12 (32.4%)	<0.001^b^
Declined to state		6 (12%)	2 (5.4%)	
Education^b^				
Elementary school		6 (12%)	1 (2.7%)	0.14
High School/GED		14 (28%)	0 (0%)	<0.001^d^
Some College		15 (30%)	4 (10.8%)	0.03
College		8 (16%)	15 (40.5%)	0.03
Graduate School		2 (4%)	15 (40.5%)	<0.001^d^
Declined to state		5 (10%)	2 (5.4%)	
Language^b^				
English proficiency		15 (30%)	35 (94.6%)	<0.001^d^
Immigrant^b,e^		50 (100%)	4 (10.8%)	<0.001^d^
Ethnicity^b^				
White		3 (6%)	16 (43.2%)	<0.001^d^
Black		6 (12%)	3 (8.1%)	0.68
Latino		26 (52%)	1 (2.7%)	<0.001^d^
Asian		7 (14%)	8 (21.6%)	0.54
Mixed		4 (8%)	4 (10.8%)	0.53
Other		4 (8%)	4 (10.8%)	0.43

^a^Data are reported as mean years ± std. deviation for age and duration of infertility

^b^Data are reported as number of patients (%) for nulliparity, multiparity, income, education level, language, and race/ethnicity

^c^T-test, two-tailed significance defined as *p <* 0.05

^d^Fisher’s Exact or *χ* ^2^, significance defined as *p <* 0.05

^e^Immigrants reported moving to the United States after 18-years old

At the LR clinic, the most common diagnosis was anovulation in 19/50 (38%), followed by tubal factor 14/50 (28%). At the HR clinic, the most common diagnoses were DOR and male factor related infertility at 14/37 (37.8%) and 19/37 (51.4%), respectively. Anovulation rate was significantly higher in the LR patient population 19/50 (38%) vs 3/37 (8.1%), *p* = 0.002. Meanwhile, the DOR was significantly higher in the HR population 14/37 (37.8%) vs 9/50 (18%), *p* = 0.006 (Table 2).

**Table 2:** Etiology of Infertility Diagnoses

Infertility diagnosis by etiology	Low resource clinic^a^	High resource clinic^a^	*P*-value
Anovulation	19 (38%)	3 (8.1%)	0.002^b^
Tubal Factor	14 (28%)	8 (21.6%)	0.09
Uterine Factor	2 (4%)	6 (16.2%)	0.107
Male Factor	8 (16%)	19 (51.3%)	0.06
Age/Diminished Ovarian Reserve	9 (18.0%)	14 (37.8%)	0.006^b^
Unexplained	6 (12%)	3 (8.1%)	0.352
Recurrent Pregnancy Loss	2 (4%)	3 (8.1%)	0.667

^a^Data reported as number of patients (% of patients)

^b^Fisher’s Exact or Chi-square analysis, significance *p* < 0.05

**Fig. 1:**
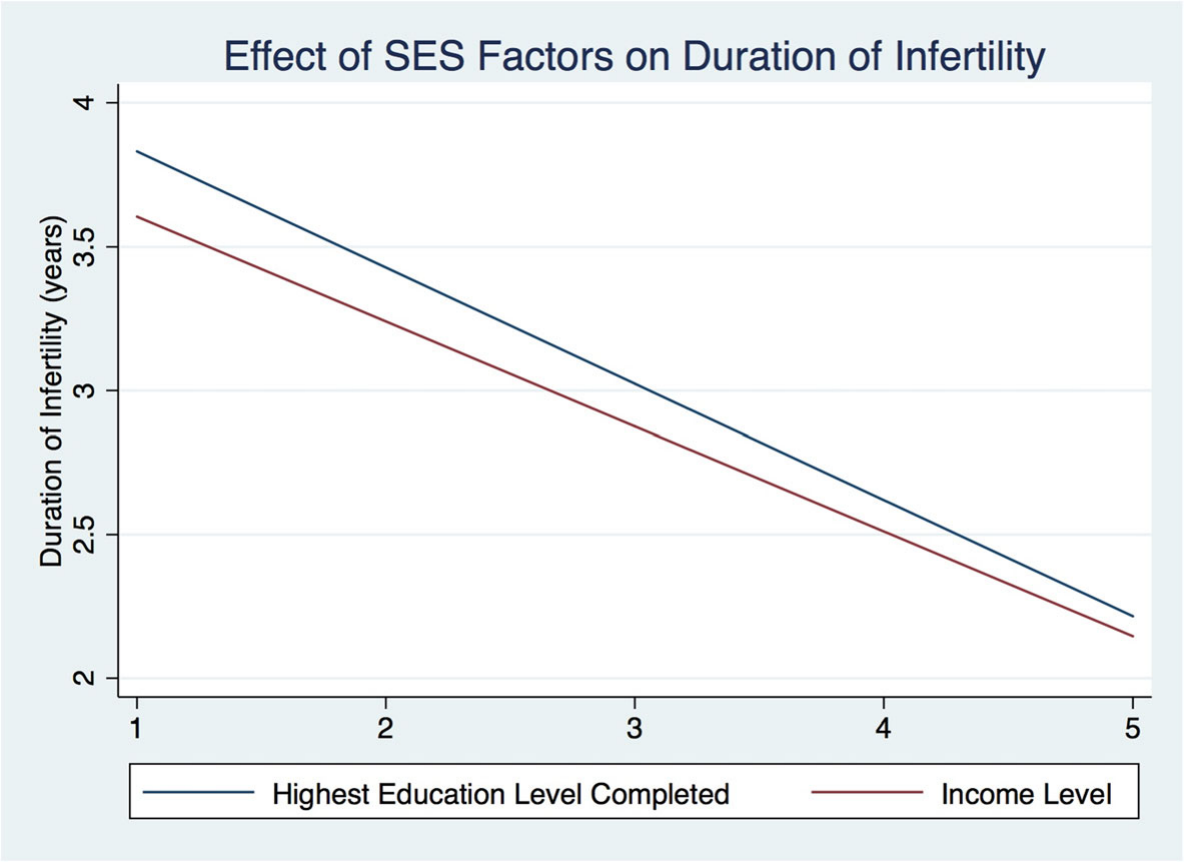
Relationship of SES Factors To Duration of Infertility. Linear fit model: relationship between education level and income with duration of infertility. *Blue*: For highest education level completed, 1 = elementary school, 2 = high school, 3 = some college, 4 = college, 5 = graduate school. R^2^ = 0.13, *p* = 0.02. *Red*: Income level, 1 = <$25,000, 2 = $25-49,999, 3 = $50,000-99,999, 4 = $100,000-199,000, 5 = > $200,000. R^2^ = 0.11, *p* = 0.04

For bivariate analyses, parity, ethnicity, and English proficiency were not significantly associated with duration of infertility in the two groups. English speakers experienced a shorter duration of infertility, though this did not quite reach statistical significance (β = −4.7, *p* = 0.06). For each advancing level of education achieved, patients presented approximately 3.5 months earlier (β = −3.5, *p* = 0.05). Patients who had gone to college presented to clinic approximately 8.4 months earlier compared to those that did not attend college (β = −8.4, *p* = 0.02). Those reporting an income ≥ $100,000 presented to clinic approximately 6 months earlier than those with an income < $100,000 (β = −6.2, *p* = 0.04). A separate analysis also showed a decreased duration of infertility with each higher income level (β = −2.3, *p* = 0.04). Patients that reported immigrating to the U.S. after age 18 presented for care 10 months later than those that did not immigrate to the US in adulthood (β = 9.8, *p* = 0.01). Among immigrants, the duration of time lived in the US was not significantly correlated with the duration of infertility. (Table 3) Fig. 1 displays the effect of education and income on duration of infertility.

**Table 3:** Impact of Sociodemographic Factors on Duration of Infertility

Variable	Adjusted β^a,c^	Adjusted *p*-value	95% CI	
Nulliparity	5.2	0.49	−9.5	20.0
English Proficiency	−4.7	0.06	−6	0.2
Immigrant Status	9.8	0.01^b^	0.2	24
Duration in US	0.005	0.99	−2.9	3.0
Income (≥$100,000/year vs. <$100,000/year)	−6.2	0.04^b^	−22	−2.8
Education (College vs. no college)	−8.4	0.02^b^	−25	−2.8
Ethnicity	2.89	0.24	−2.1	7.8
White	−7.3	0.35	−23.3	0.32
Black	13.0	0.43	−20.1	46.1
Latino	−4.3	0.58	−19.9	11.3
Asian	14.4	0.12	−4.0	32.8
Mixed	−5.2	0.73	−35.4	25.1
Other	8.6	0.68	−32.9	50.2

^a^ Linear regression model used controlling for age at the start of pregnancy attempt. Women with prior infertility treatments were excluded from the analysis (*n* = 2)

^b^ Significance defined as *p <* 0.05

^c^ β represents duration of infertility in months (where a negative value represents less months, and a positive value represents more months)

For the multivariable analysis, we controlled for English proficiency, immigrant status, income level, education level, and age at the initiation of pregnancy attempt. Interestingly, only education level had a significant effect on duration of infertility. Patients attending college presented for care about 8 months earlier compared to those that did not attend college (β = −8.3, *p* = 0.049), when controlling for other SES factors. (Table 4)

**Table 4:** Multivariable Analysis of SES Factors on Duration of Infertility

Variable	Adjusted β^a^	Adjusted *p*-value	95% CI	
English Proficiency	−4.8	0.73	−21.9	31.6
Immigrant Status	12.6	0.6	−10.0	36.0
Income (≥$100,000/year vs. <$100,000/year)	−6.0	0.6	−21.2	37.5
Education Level (College vs. no college)	−8.3	0.049^b^	−32	−2.0

^a^ Multivariable linear regression model. β represents duration of infertility in months (where a negative value represents less months, and a positive value represents more months)

^b^ Significance defined as *p <* 0.05

## Discussion

Despite the presence of wide disparities, there has been slow progress towards addressing unmet needs in the field of infertility. In 2001, the WHO declared that infertility should be considered a global health problem, and eight years later officially defined infertility as a disease of the reproductive system [7, 22]. Recently, ASRM released an Ethics Committee Opinion calling members to recognize and address disparities in access to infertility care [23].

Describing the causes of infertility in a low resource patient population is a critical first step when thinking about resource allocation and ways to address the needs of patients. In the developing world, particularly in Africa, studies have been published detailing the causes and burden of infertility, most of which note that tubal factor is the most common diagnosis, with a reported prevalence ranging from 33-85% [24, 25]. These studies also detail the devastating effects of childlessness, which have prompted efforts to discover and implement innovative and cost-effective solutions [24, 26–28]. Much less is known about the demographics and experience of infertility as it presents across socioeconomic domains in the US. Our study is the first that seeks to characterize the causes of infertility in a low-resource, largely immigrant population presenting for infertility care in the US.

### The need for ART

In our study, infertile women from the LR group were younger in age and report a longer duration of infertility compared with the HR group. In LR patients, the most common diagnosis was anovulation. The leading etiology of anovulatory related infertility in women is polycystic ovary syndrome (PCOS) [29]. In many patients with PCOS, interventions such as weight loss and ovulation induction (OI) are effective initial interventions [30]. Costs can be limited by using affordable interventions such as OI with letrozole or clomiphene citrate or laparoscopic ovarian drilling [31]. These approaches are cost-effective methods for treating infertility that do not require the infrastructure of a high complexity laboratory [32]. It should be noted that basic infertility services are not covered under most county, state and federal public health programs.

Studies in developing countries have found the most common diagnosis for infertility is tubal factor due to sexually transmitted illnesses (STI) [24, 25]. In LR patients, tubal factor was the second most common diagnosis at 28%. An affordable option for some of these patients is tubal surgery but success rates vary widely and range from 0-22% for poor prognosis and 58-77% for good prognosis patients [33]. Tubal surgery outcomes likely vary based on provider experience and are not always covered by insurance or offered by infertility specialists. Traditionally, the approach to combating infertility in low-resource settings has focused on prevention and treatment for STIs, preventing postpartum infection, and making abortion practices safer [34]. These interventions still leave childless couples with limited options and do not prevent many forms of infertility. In 2007, the European Society for Human Reproduction and Embryology started a task force on infertility in low-resource settings, which encouraged the development and investigation of low cost ART protocols [28]. Such programs, which incorporate use of lower dose hormonal stimulation and/or simplified lab approaches, have been successful at achieving live births but, at present, remain with limited experience [35, 36].

The LR patients are younger than many of those seen in fee-for-service clinics, and generally have a good prognosis for a live birth if given the opportunity to be treated with ART. Similar to the developing world, women with insufficient income to pay for ART services experience an insurmountable gap in access to care. The effects of this are distressing, as many of these women tie a significant part of their identity and livelihood to the ability to produce biological children [15]. Since the US is a resource rich setting, it should be feasible to find solutions towards addressing these inequities.

### High ART costs limit accessibility

In an economic analysis, Chambers et al. describe the high entry cost for IVF [9]. In other developed countries where the cost per IVF cycle is lower, there was a higher utilization of ART services. In the US, some legislative and administrative steps have been made towards increasing ART accessibility. Fifteen states have laws requiring insurance companies to cover some aspect of infertility treatment, but some definitions of infertility are overly strict and IVF is often not covered [37]. While the Affordable Care Act brought some improvements in access to preventative maternal and gynecological care, infertility treatments are excluded [38]. Even in states with mandated coverage of IVF, patients under federal, state or county safe-net programs do not have this benefit. In the US, price is a barrier that separates those that are able to pay for standard of care treatment vs those that must accept substandard or no care in many cases. In our study, length of time lived in the US did not affect the duration of infertility in recent immigrants. This suggests that regardless of how long immigrants spend living and working in the US in adulthood, infertility services remain inaccessible for the underserved. Immigrants may also face challenges such as obtaining loans or credit in order to pay for infertility treatments. Additionally, physicians or mid-level providers may not be aware of the prevalence and burden of infertility in this population, leading to delays in workup or referrals. Provider allocation bias due to implicit assumptions about ethnicity or income may also influence referral for infertility services, as seen in other areas of health care [39]. Although the economic barrier is high, earlier referrals may allow some patients to have the time to save enough money for an IVF cycle.

### Education level impacts access to infertility care

Aside from cost, the ability to navigate the health care system also poses a challenge for LR patients. In our cohort, bivariate analysis showed that lower education level, income, and immigrant status were associated with a longer duration of infertility. This is consistent with previous work by Smith et al. in which, even among HR infertility patients, education and income were significantly associated with access to reproductive services [40]. It is important to note that while English proficiency did not significantly impact the duration of infertility in our study, findings trended towards significance. This was likely due to low numbers, and we acknowledge that the language barrier impacts ability to access health services. Interestingly, in the multivariable analysis, education was the only significant factor affecting duration of infertility. While education, income, immigrant status, and English proficiency are interwoven, education appears to be an independent factor enabling patients to seek care for their infertility. Studies show that patients with less education are likely to have lower health literacy, which has been linked with decreased use of preventative health services and delay in diagnoses [41, 42]. Decreased health literacy also impacts patients’ ability to adhere to instructions, leading to worse medical outcomes [43, 44]. To address these issues, clinics should provide patient information resources that take into account different levels of health literacy [45]. Additionally, awareness and education for primary providers to recognize and screen for infertility may facilitate more timely referrals for infertility workup and care.

### Strengths and limitations

Strengths of this study are that we highlighted the demographics and causes of infertility of a patient population that is understudied in the infertility literature. Limitations include a small sample size, though the largest analysis of this population in the literature, and the lack of long-term outcome data. The small sample size may have precluded significant findings on the effects of sociodemographics on duration of infertility in our bivariate and multivariable analyses. Additionally, this study takes place in a single geographic location and an academic setting, which may be more diverse than a typical private fertility clinic in the US. Ethnicity or English proficiency may have significantly affected duration of infertility of there was a larger sample size or if the population was more homogeneous. Another limitation is that reported duration of infertility is a not a direct metric of barriers to infertility care. Other areas of health disparities research demonstrate a delay in access to preventative health services by uninsured patients [46, 47]. We believe that the duration of infertility mirrors this delay and is a reasonable proxy for difficulty in accessing health services.

## Conclusions

We conclude that women seeking infertility care in a low resource urban setting are younger and face many challenges to obtaining care. This group is one that is understudied, despite the unmet demand for infertility care. We hope that drawing attention to the need for infertility services in an under-resourced population will help drive more resources towards increasing ART access. Larger scale studies will provide a framework of knowledge to further address the impact and inequities in access to infertility care in this vulnerable population.

## Additional file

Additional file 1: Social Demographics Assessment Survey. (DOCX 67 kb)

## Abbreviations

AFC: Antral follicle count
AMH: Antimüllerian hormone
ART: Assisted reproductive technology
DOR: Diminished ovarian reserve
ESHRE: European society for human reproduction and embryology
FSH: Follicle stimulating hormone
GED: General educational development (high school equivalent degree)
HR: High resource
IVF: In vitro fertilization
LR: Low resource
OI: Ovulation induction
SES: Socioeconomic status
SFGH: San Francisco General Hospital
UCSF: University of California San Francisco
