# Supplementary material for: Demographic analysis of a low resource, socioculturally diverse urban community presenting for infertility care in a United States public hospital
Source: Contracept Reprod Med. 2017 May 3;2:17. doi: 10.1186/s40834-017-0044-7 (PMC5683225; doi:10.1186/s40834-017-0044-7)
Supplement: Additional file 1: — Social Demographics Assessment Survey. (DOCX 67 kb) [file 40834_2017_44_MOESM1_ESM.docx]

**Appendix 1**

**Social Demographics Form**

(1) What is your gender?

- - Male
  - Female

(2) In what year were you born? _____________

(3) In what country were you born? ________________

(4) How long have you been in the United States? __________

(5) What do you consider your ethnicity to be? (Check all that apply)

- - White/European American/ Caucasian
  - African-American/Black
  - Latino/Hispanic
  - Asian
  - Pacific Islander
  - Native American, American Indian, Alaskan Native or Indigenous
  - Mixed/Multi-ethnic: Please specify if possible: ______________
  - Do not know

(6) What is the primary language spoken at home?

- - English only
  - Spanish Only
  - Mandarin
  - Cantonese
  - Vietnamese
  - Arabic
  - Tagalog
  - Korean
  - Bilingual: Which languages?______________
  - Other: _______________

(7) What is your religion?

- - Protestantism
  - Catholicism
  - Christianity
  - Judaism
  - Atheism/agnosticism
  - Buddhism
  - Islam
  - Other:_________________

(8) What is your marital status?

- - Married
  - Unmarried (single, never married, domestic partner)

(9) Number of total years married? ________

(10) Number of previous marriages (not including current marriage)? ________

(11) Number of children from previous marriages? ______

(12) Have you been pregnant before?

- - Yes
  - No

(13) If yes, how many times? _____

(14) How many children do you have? ______

If you have children:

Age of children: _____

Gender of Children:

- - Male
  - Female
  - Other_________

(15) What is the highest degree or level of school you have completed?

- - Grade school
  - High School, GED
  - Some college
  - College degree
  - Graduate degree

(16) What is your current or previous Occupation?

- - Administrative/Technical
  - Clerical/Sales
  - Skilled Labor
  - Unskilled Labor
  - Homemaker

(17) What is your total household income?

- - Less than $25,000
  - $25,000-$50,000
  - $50,000-$75,000
  - $75,000-$100,000
  - $100,000-$150,000
  - $150,000-$200,000
  - $200,000-$250,000
  - More than $250,0000

(18) What best describes your medical insurance?

- - Private Health Insurance
  - Public Health Assistance Program (including, for example, Healthy San Francisco)
  - No Health Insurance
  - Other

(19) Does your insurance cover fertility care for either diagnosis or treatment?

- - Yes
  - No
  - I am not sure

(20) Have you previously sought any form of infertility treatment?

- - Yes
  - No
  - I am not sure

(21) What type of infertility treatment have you previously had?

- - Medications
  - Medications with intrauterine insemination
  - In Vitro Fertilization (IVF)
  - Other form of treatment
  - No treatment

(22) If other form of treatment selected above, what type of treatment:_____________________

(20) Please indicate if you have had any of the following (check all that apply).

Ectopic pregnancy ______________times

Therapeutic Abortion _____________times

Miscarriage (spontaneous abortions)_____________times

Stillbirth ____________times
